# Gene–Environment Interactions and Intermediate Phenotypes: Early Trauma and Depression

**DOI:** 10.3389/fendo.2014.00014

**Published:** 2014-02-17

**Authors:** Orla P. Hornung, Christine M. Heim

**Affiliations:** ^1^Institute of Medical Psychology, Charité University Medicine Berlin, Berlin, Germany

**Keywords:** depression, stress, maltreatment, development, HPA axis, polymorphisms

## Abstract

This review focuses on current research developments in the study of gene by early life stress (ELS) interactions and depression. ELS refers to aversive experiences during childhood and adolescence such as sexual, physical or emotional abuse, emotional or physical neglect as well as parental loss. Previous research has focused on investigating and characterizing the specific role of ELS within the pathogenesis of depression and linking these findings to neurobiological changes of the brain, especially the stress response system. The latest findings highlight the role of genetic factors that increase vulnerability or, likewise, promote resilience to depression after childhood trauma. Considering intermediate phenotypes has further increased our understanding of the complex relationship between early trauma and depression. Recent findings with regard to epigenetic changes resulting from adverse environmental events during childhood promote current endeavors to identify specific target areas for prevention and treatment schemes regarding the long-term impact of ELS. Taken together, the latest research findings have underscored the essential role of genotypes and epigenetic processes within the development of depression after childhood trauma, thereby building the basis for future research and clinical interventions.

## Introduction

Mood disorders are among the most prevalent forms of mental illness. The lifetime prevalence of major depressive disorder (MDD) among US adults is 16.5%, with women being 70% more likely than men to develop depression. Stress and trauma exposure are major risk factors for the development of depression, particularly when experienced early in life. However, up to 40% of the risk to develop depression appears to be genetic. Additional risk factors of depression include family history of depression, past episodes of depression, and specific personality traits, such as neuroticism among others. The combined impact of genetic and environmental factors contributing to the pathogenesis and onset of depression is in line with the diathesis–stress model (1). According to this psychological model, the interaction between predispositional factors and stressors leads to disorders such as depression.

A wealth of studies document the prominent role of early life stress (ELS), such as sexual, physical or emotional abuse, emotional or physical neglect, or parental loss, in the pathogenesis of depression [see Ref. (2–5)]. During periods of heightened neural plasticity throughout development, brain regions involved in the regulation of emotion and the mediation of the stress response appear to be particularly sensitive to the effects of ELS. Such experience-dependent plasticity may produce altered neural circuits, and hence maladaptive responsiveness to the environment, that ultimately leads to elevated risk for depression. However, not all individuals who experience ELS develop depression in adulthood, underscoring the importance of investigating the underpinnings of such variability in the link between ELS and depression. Understanding the source of such outcome variability could aid in identifying pre-disease vulnerability tests and in devising targeted prevention strategies.

A major body of evidence has rapidly accumulated over the past years concerning interactive effects of genetic factors and ELS in determining depression risk (3). These studies have mostly focused on studying the moderating effects of variations in candidate genes acting in neurobiological systems that have been implicated in the pathophysiology of major depression in large-scale epidemiological samples. Outcome measures usually comprise lifetime or current diagnoses of major depression or dimensional ratings of depressive symptoms, respectively. More recently, however, this focus has shifted to consider Gene × ELS interaction effects on phenotypic features, such as neural structure or function, neuroendocrine function, or behavioral constructs, that may mediate the link between stress and clinical manifestations of depression and that may be affected by genetic variation [see Ref. (6)]. This so-called intermediate phenotype approach is thought to provide more potent and valid associations between genetic factors and a given disease, such as depression, because genes are more proximate to the pathophysiological pathways that lead to depression than to the diagnostic categorization as defined by DSM-IV. In the following, we provide an overview of studies identifying interactions between candidate genes and ELS in the prediction of the clinical manifestation of depression as well as corresponding intermediate phenotypes.

## Gene × ELS Interactions in the Prediction of Depression

A large body of research has focused on identifying candidate genetic variations that interact with ELS in predicting current or lifetime diagnoses, or symptom severity, of MDD. Core symptoms of depression include sad mood, loss of pleasure, sleep disruption, loss of appetite, decreased libido, impaired cognition, feelings of worthlessness and guilt, and suicidal ideation. Diagnosis of an episode of MDD requires the presence of five or more symptoms over a 2-week period, with sad mood or loss of pleasure being one of the symptoms. Candidate genes studied to date have considered single nucleotide polymorphisms (SNPs) in neurobiological systems relevant to stress and depression, i.e., the monoaminergic neurotransmitter, corticotropin-releasing hormone (CRH), and glucocorticoid receptor (GR) and GR-chaperone systems, as well as neurotrophin, oxytocin, and endocannabinoid systems.

### Serotonin transporter

The serotonergic system is the most prominent target of antidepressant pharmacotherapy [see Ref. (1)]. The main mechanism of action of serotonergic drugs is based on the inhibition of serotonin reuptake. Serotonergic neurons originate in the nucleus raphé and project to the forebrain. Serotonin is thought to be critical for regulating emotional responses in cortical–limbic networks and has regulatory effects on neuroendocrine stress responses. Stress mediators, such as CRH, modulate serotonin release in the nucleus raphé and regulate serotonin release in the medial prefrontal cortex (PFC) (7). Based on this evidence, the serotonergic system is of interest in the study of Gene × ELS interactions in predicting MDD. At the synaptic level, serotonergic neurotransmission and reuptake is regulated by the serotonin transporter gene (*SLC6A4*). A common functional polymorphism (5-HTTLPR) in the promoter region of *SLC6A4* involves either short (s) or long (l) alleles, with the short allele variant being associated with lower transcriptional efficiency. In a seminal report, Caspi et al. (8) demonstrated in the prospective Dunedin Birth Cohort of New Zealand that 5-HTTLPR interacts with stressful life events, including child maltreatment, to predict current and lifetime diagnoses of MDD as well as suicide attempts. Specifically, the s-allele was found to confer risk for developing depression in the context of life stress in this study. The report by Caspi et al. (8) served as an impetus for a wealth of subsequent studies, providing both replications and failures to replicate the original finding [see Ref. (9)]. A meta-analysis failed to confirm the interaction effect (10). A critical debate over this topic identified potential sources of this heterogeneity, mainly stemming from methodological differences between studies (11). The method of assessing ELS, as well as the type and timing of ELS, appear to be important effect modifiers (12). In addition, the definition of the outcome variable seems to be of particular importance. Specifically, the 5-HTTLPR × ELS interaction appears to predict persistent depression (i.e., an episode of MDD diagnosed at least twice), but is not predictive of a single-episode of MDD (13). Furthermore, it has now been reported that the interaction between 5-HTTLPR and ELS in predicting depression is moderated by other polymorphisms (see below).

In the light of a female preponderance in the risk for MDD, sex differences in Gene × ELS interactions have been studied. An interaction of 5-HTTLPR and ELS in predicting depression was found to be more pronounced in females than in males (14, 15). Carli et al. (16) reported that the l-allele was associated with increased risk for depression in male subjects exposed to ELS. It remains unclear whether these Gene × ELS × Sex interactions are attributable to sex differences at a neurobiological level or to sex differences in exposure variables. In addition to sex differences, age may moderate the 5-HTTLPR × ELS interaction effect in the prediction of depression, e.g., the l-allele carrying genotype exhibits higher vulnerability to develop depression in interaction with ELS in elderly adults (17).

### Dopamine receptor (DAT1)

Dopamine is assumed to play an important role in the pathogenesis of MDD through its function in regulating motivation, reward, and the ability to experience pleasure (18). Stress contributes to loss of pleasure or anhedonia, a cardinal symptom of depression, by impacting on dopaminergic pathways that are implicated in reward processing, such as the mesocorticolimbic pathways (19, 20). In a study of male adolescents, a polymorphism, rs40184, in the dopamine receptor gene (*DAT1*) interacted with perceived maternal rejection in predicting the presence of MDD and suicidal ideation (21). This Gene × ELS interaction effect was specific to the prediction of MDD and did not predict anxiety, perhaps in accordance with the behavioral effects of dopaminergic pathway dysfunction. Of note, polymorphisms in 5-HTTLPR and other genes appear to interact with stress in predicting reward learning, which is an important intermediate phenotype in the link between genes, stress, and depression (see below).

### Corticotropin-releasing hormone receptor and binding globulin

The central CRH system plays a pivotal role in the relationship between ELS and depression. CRH neurons in the paraventricular nucleus of the hypothalamus form the central component of the HPA axis, the major neuroendocrine stress response system in mammals. Cortisol, the end product of the HPA axis exerts stress-adaptive metabolic, immune-regulatory and behavioral effects, and controls HPA axis responsiveness via negative feedback inhibition. In addition, CRH acts in a widespread circuitry connecting cortical, limbic, and brainstem regions and is thought to integrate endocrine, autonomic, and behavioral responses to stress. Behavioral effects of CRH resemble the core signs and symptoms of stress, depression, and anxiety. These effects are mediated though CRHR1 receptors (22). CRH hyperactivity is a core biological feature of MDD (23). There is ample evidence from animal and human studies that ELS contributes to such hyperactivity (24). Hence, the CRHR1 gene has been studied as a candidate gene in moderating the relationship between ELS and MDD. Bradley et al. (25) demonstrated an interaction between self-reported childhood abuse and polymorphisms in the CRHR1 gene in predicting adult depression. Two haplotypes were identified that conferred protection against developing depression in persons who reported moderate–severe child abuse exposure, as measured by the Childhood Trauma Questionnaire (CTQ) (26). When exposed to moderate–severe childhood abuse, persons who were homozygous for the protective haplotypes showed significantly less symptoms of depression as compared to individuals with other haplotype combinations. Within the protective haplotypes, rs110402 was most potent in predicting depression after ELS, with A allele carriers being protected. These findings were replicated in two independent cohorts (25, 27). In the Polanczyk et al. (27) study, the interaction was replicated in the UK Environmental Risk Longitudinal Twin Study using retrospective self-reports of childhood maltreatment, assessed by CTQ similar to the Bradley et al. (25) study. In contrast, these authors could not replicate the interaction effect in the Dunedin Birth Cohort that provides prospective multi-informant reports of childhood maltreatment. The authors, therefore, speculate that the CRHR1 gene moderates emotional memory, which in turn may be linked to risk versus resilience against depression (27). Other potential reasons for discordant results might be differences in features of ELS. Heim et al. (22) reported that the interaction between rs110402 and ELS in predicting depression was specific to physical abuse. This produced a sex difference in the interaction of rs110402 and ELS, because males were more frequently exposed to physical abuse than females in this sample. Of note, Grabe et al. (28) also report interactions between different SNPs in CRHR1 and specifically physical neglect, as measured by CTQ. CRHR1 polymorphisms also interact with physical assault during childhood or adolescence in predicting suicide risk particularly in males (29), further stressing the importance of this gene in interacting with physically abusive experiences. These results suggest that the environmental exposure must be carefully assessed in Gene × ELS studies [see Ref. (3)]. In addition, two recent studies have demonstrated that CRHR1 SNPs interact with 5-HTTLPR in predicting depression after ELS (30, 31). Such three-way Gene × Gene × ELS interaction effects may contribute to inconsistent results between studies considering only one gene. Beyond CRHR1 polymorphisms, an interaction of a polymorphism in the CRH binding protein (CRHBP) gene, which may modulate the availability of CRH at the synapse, and childhood trauma in predicting suicide attempts has been recently reported (32). Taken together, these studies support a pivotal role of the central CRH system in mediating the link between ELS and depression, and the genetic moderation of this link.

### Glucocorticoid receptor and FKBP5

The regulating effects of cortisol on brain and behavior are mediated by binding of the hormone to specific receptors. High-affinity mineralocorticoid receptors (MR) are located in the hippocampus and exert tonic inhibitory effects on basal HPA axis activity, whereas high-affinity GR are widely distributed throughout the PFC, amygdala, hippocampus, and brainstem, and are critical to regulating responses during conditions of elevated cortisol secretion or stress. Upon binding of cortisol to the intracellular cytosolic GR, the GR–hormone complex becomes activated and translocates to the nucleus, where the complex binds to gene responsive elements which impact on transcription of proteins. Relative GR resistance is a core feature of MDD, perhaps leading to disinhibition of central CRH secretion and HPA axis hyperactivity. The GR gene has been studied as a candidate gene that may moderate the link between ELS and MDD in one study. Bet et al. (33) demonstrated in a longitudinal aging study conducted in the Netherlands in individuals aged 55–85 years that the 22/23EK and 9beta polymorphisms in the GR gene interacted with life events and adversities during youth, including war experiences, sexual abuse, parental loss, or physical illness in the prediction of clinically relevant depression.

The sensitivity of the GR is fine-tuned by FK506 binding protein 51 (FKBP5), a co-chaperone of hsp90. When bound to the GR-hsp90 complex, FKBP5 decreases GR affinity for cortisol and prevents translocation of the GR to the nucleus [see Ref. (34)]. Of note, glucocorticoids induce the expression of FKBP5, forming an ultra-short negative feedback loop for GR activity (34). Given the important role of GR in regulating stress responses and the evidence for GR resistance in depression, variation of the FKBP5 gene likely is a critical modulator of the relationship between ELS and depression (34). Variants in the FKBP5 gene have been shown to modulate risk of developing PTSD in relation to childhood trauma (35). In a recent population-representative sample of more than 2000 German people, Appel et al. (36) report an interaction between rs1360780 of the FKBP5 gene and ELS, assessed by CTQ, in predicting both depressive symptoms and DSM-IV diagnoses of MDD. Specifically, TT carriers who reported physical abuse were at greater risk for depression than CC/CT carriers exposed to physical abuse. This interaction effect was confirmed for individuals who experienced very severe forms of sexual and emotional abuse (36). Another recent study in 884 adolescent and young adult individuals confirmed an interaction of different FKBP5 variants, including rs1360780, and traumatic life events in predicting the onset of MDD in a 10-year prospective study (37). These latter findings were replicated in the UK Environmental Risk Longitudinal Twin Study (37). FKBP5 polymorphisms were further reported to interact with CTQ score in predicting suicide attempts in an adult African American sample (38). The same group reported an additive effect of FKBP5 polymorphism on the interaction of the CRHBP gene and ELS in predicting suicide attempts (32). These results, taken together, strongly implicate the FKBP5 gene in the pathogenesis of stress-related depression, likely mediated through determining individual level of GR resistance and, consequently, glucocorticoid signaling.

### Brain-derived neurotrophic factor

Brain-derived neurotrophic factor (BDNF) is a widely expressed neurotrophin in the brain that is implicated in neuronal growth, synaptic plasticity, and neuronal survival, and therefore is thought to play an important role in structural brain abnormalities observed in depressed individuals, such as reduced hippocampal volume (1, 39). In animal models, prolonged stress exposure and elevated glucocorticoid levels down-regulate BDNF expression, whereas administration of antidepressant drugs induces BDNF expression; antidepressant treatment normalizes hippocampal volume in depressed patients (1). The BDNF gene contains a functional polymorphism (rs6265), which is associated with a valine to methionine substitution (Val66Met) and leads to reduced BDNF expression. Aguilera et al. (40) report an interaction of this polymorphism and childhood sexual abuse in the prediction of adult depression. Specifically, carriers of the Met allele (Met/Met, Met/Val) who reported childhood sexual abuse were at greater risk for developing depressive symptoms compared to carriers of the Val/Val genotype. Several studies have provided evidence that the 5-HTTLPR and BDNF Val66Met polymorphisms interact in predicting depression after ELS. In one study, maltreated children carrying the Met allele of BDNF Val66Met and two short alleles of 5-HTTLPR exhibit highest depression scores (41). This BDNF Val66Met × 5-HTTLPR × ELS interaction in the prediction of depression was replicated in a study of adult female twins (42). However, two other studies failed to replicate this three-way interaction in the prediction of depression (40, 43), perhaps suggesting further moderating factors.

### Oxytocin receptor

The central oxytocin system mediates social attachment, including mother–infant bonding, and buffers emotional and physiological responses to stress (44). For example, intranasal administration of oxytocin reduces amygdala activation in response to fearful visual stimuli in humans (45). In patients with MDD, plasma oxytocin concentrations are inversely correlated with symptom severity of depression and anxiety (46). Women reporting exposure to childhood maltreatment exhibit deceases in cerebrospinal fluid oxytocin concentrations, which in turn were associated with increased anxiety (22). In a mouse model, oxytocin exerted anxiolytic effects by regulating serotonin release though activation of oxytocin receptors (OXTR) in serotonergic neurons (47). Therefore, the OXTR receptor gene is a candidate gene that may moderate the link between ELS and depression. One study reported an interaction of OXTR rs2254298 and adverse parental environment in predicting symptoms of depression in 9–14 years old girls (48). Girls who were heterozygous (A/G) for the *OXTR* rs2254298 polymorphism and reported high levels of adversity exhibited the most severe depression as well as physical symptoms and social anxiety. Of note, the level of parental adversity in this study was operationalized as a history of recurrent MDD in the mother, hence measured associations between adversity and depression in the girls may also reflect genetic risk. Other studies report associations of another OXTR polymorphism, rs53576, on several intermediate phenotypes that are relevant in the link between ELS and depression, which are reviewed below.

### Endocannabinoid receptor

The endocannabinoid system, consisting of endocannabinoid receptors and their endogenous ligands appear to play a role in the adaptation to stress and emotional responses (49). Endocannabinoid receptor 1 receptor knockout in mice modulates the effects of chronic unpredictable stress on the development of anhedonia in mice (50). In a recent study Agrawal et al. (51) evaluated the role of a polymorphism in the human endocannabinoid receptor (CNR1), rs1049353, in moderating the association between childhood physical abuse and anhedonia or MDD in young adult US women. Those women who carried two copies of the minor allele (G/G) exhibited less anhedonia when exposed to childhood physical abuse compared to carriers of the A allele (A/A or A/G). This protective effect was also seen for MDD, but was largely attributable to anhedonic MDD. The effect was replicated in an independent Australian sample (51). Of note, the interaction effect was specific to physical abuse and was not observed in relation to sexual abuse.

### Summary

In summary, a number of genes operating in neurobiological systems that mediate stress responses, social–emotional regulation, and/or synaptic plasticity have been identified to date as significant moderators of the relationship between ELS and depression. While several of these interaction effects have been replicated across independent studies, there is heterogeneity in the field. It becomes evident that multiple factors, both on the genetic and on the environmental side of the equation, must be considered in the prediction of depression. First, it is unlikely that a single gene is implicated in the relationship between ELS and depression and it appears that multiple polymorphisms in different genes interact with the environment in determining depression risk. Second, early environmental exposures such as ELS are complex in nature and there is increasing evidence for specificity of Gene × ELS interaction effects in depression, inasmuch as effects were reported for one type of maltreatment but not another. In addition, the method of assessment of ELS (i.e., retrospective self-report versus prospective informant reports) influences results. Additional sources of variability concern gender, ethnic background, and resources such as social support, among others. Future studies should scrutinize these complex interactions. In addition, genome-wide association studies (GWAS) of Gene × ELS interactions in depression have the potential to confirm candidate gene effects and/or to reveal novel genes that might be critical in the link between ELS and depression that were not selected as candidates based on theoretical reasoning [for detailed discussion, see Ref. (3)]. Finally, the functional significance of many of the identified polymorphisms remains unknown and, hence, the biological mechanisms that mediate genetically determined risk or resilience to depression, in the context of ELS, are poorly understood. Toward that end, there is an increasing body of studies adopting the so-called intermediate phenotype approach to further our understanding of the association between ELS and depression, as discussed in the following.

## Gene × ELS Interactions in Depression: Concept of Intermediate Phenotypes

Genetic disposition and environmental exposures are linked to the manifestation of a psychiatric syndrome through a number of intermediate steps that span from altered cells to altered neural circuits to altered behavioral domains, such as cognition or emotion processing. The intermediate phenotype approach in psychiatry seeks to identify genetic effects on each level of these intermediate phenotypes, which are more proximal to the etiological process than the diagnosis itself that is derived from disputable classification criteria. By mapping neurobiological effects of a risk gene on known brain phenotypes of a disorder, this approach also allows for deciding whether a risk gene indeed is relevant for a pathophysiological mechanism involved in the given disorder or whether a found association between a gene and a disorder is a pure epiphenomenon of another causal association. Hence, the intermediate phenotype approach increases power and validity of detecting a genetic effect on a psychiatric disorder (6, 52). While a number of intermediate phenotype studies have been conducted to test for genetic main effects on phenotypes linked to a given disorder, only a few studies have applied this approach to elucidate Gene × ELS interactions in depression. In order to study Gene × ELS effects on intermediate phenotypes that might be located on the pathway to the manifestation of depression, it must be decided which phenotypes are indeed relevant for mediating the link between ELS and depression risk. In other words, some of the known biological features of depression might be linked to ELS, whereas others are not. Therefore, the selection of the appropriate intermediate phenotype for Gene × ELS studies needs to be informed by research on the contribution of ELS to the neurobiology of MDD.

## Intermediate Phenotypes Linking ELS and MDD

A number of clinical studies have now elucidated effects of ELS on the neurobiology of MDD in humans [see Ref. (3, 53)]. Adult women with histories of childhood sexual or physical abuse exhibit markedly increased neuroendocrine and autonomic responses to psychosocial laboratory stress, particularly those with depression (54). Other alterations in humans with ELS experiences altered glucocorticoid feedback, increased levels of inflammation, increased central CRH activity, and decreased activity of the prosocial neuropeptide, oxytocin (22, 55–59). A small hippocampus has also been linked to ELS in humans (60–65). ELS also has been linked to reduced volumes of the insula (61), orbitofrontal cortex (61, 66), anterior cingulate (61, 67), caudate (61), and medial PFC (62, 68). Moreover, increased amygdala volume and reactivity (61, 69–71) as well as left basal ganglia dysfunction in response to reward cues (72) have been reported as a function of ELS. Furthermore, ELS is associated with altered cortical affective processing in patients with various psychiatric disorders (73). It appears that ELS produces changes in a connected neural network that fails to adapt or compensate in response to additional challenge, thereby leading to the behavioral and physiological changes that ultimately form the syndrome of clinical depression (59). More recently, epigenetic marks that occur as a function of ELS have been reported, such as hyper-methylation of the GR gene, resulting in reduced GR expression (74, 75). In a genome-wide post-mortem study of hippocampal tissue obtained from suicides, ELS was associated with differential methylation of 362 genes, particularly affecting genes involved in neuronal plasticity (76). In several of the above studies, neurobiological changes clearly distinguished between depressed persons or suicide victims with and without ELS experiences, including HPA axis reactivity (54, 59), hippocampal volume (65), PFC volume (62), amygdala reactivity (69), and methylation pattern (74, 76), suggesting that these phenotypes mediate the link between ELS and MDD and, therefore, might be appropriate targets for studying Gene × ELS interactions in depression. ELS further modulates behavioral domains, including social–emotional processing (71, 77), conditioned fear (78), or reward anticipation (72), all of which represent relevant intermediate phenotypes for Gene × ELS studies in depression.

## Gene × ELS Interaction Effects on Intermediate Phenotypes Related to Depression

Only a small, but rapidly increasing number of studies to date have assessed the combined impact of genetic variation and ELS exposure on intermediate phenotypes of interest for the development of depression. In addition to studies in humans, several studies in non-human primates provide additional insight into Gene × ELS interactions in predicting intermediate phenotypes relevant to depression.

### Gene × ELS interaction effects on the HPA axis

Due to the central role of the HPA axis activity in the pathogenesis of depression, it is of interest to investigate Gene × ELS interaction effects on HPA axis activity as intermediate phenotype of depression. The 5-HTTLPR polymorphism of the serotonin transporter gene (SLC6A4) has been of prime interest in this context with the s-allele being associated with an elevated risk for depression following critical life stress compared to the l-allele (8). Several studies indicate that there is an interaction between 5-HTTLPR genotype and ELS on HPA axis activity.

In a laboratory setting with a standardized stress task (stress induced by a subtraction task and semi-structured interview) girls aged between 9 and 14 years showed higher and prolonged levels of cortisol if they were s-allele carriers compared to l-allele carriers (79). These findings support the idea that HPA axis activity is differentially affected by an interaction between 5-HTTLPR genotype and stress. Adding to these findings, s-allele carriers with a history of stressful life events showed increased cortisol reactivity to laboratory stress (public speaking) in a sample of healthy male adults (80). Genotype and life stressor history, therefore, both seem to modulate endocrine reactivity to acute stress. Young adults who had taken part in the Trier Social Stress Test (TSST) showed distinct cortisol reactivity depending on the evaluative context of the condition (critical, supportive, or none) (81). Only in the negative audience condition an interaction with the 5-HTTLPR genotype emerged indicating that the s-allele carriers are particularly sensitive to social threat and therefore show heightened endocrine stress responses.

An age-dependent pattern of results was found in a recent study (82). HPA axis activity was investigated by measuring cortisol responses and stressful life events in children, young and old adults who were subjected to the TSST. An interaction between 5-HTTLPR genotype and life stress on endocrine stress responses was found only in young adults and only if the life stressors had occurred in the first 5 years of life suggesting that the first years of life are particularly sensitive. An interactive effect between 5-HTTLPR genotype and stress was also found in cortisol responses of newborn babies exposed to painful stimulation (83). Heightened sensitivity of s-allele carriers toward stress exposure therefore seems to be present at an early developmental stage.

There is evidence for a Gene × Gene × ELS effect; an interaction between 5-HTTLPR and acute stress (TSST) on cortisol response emerged in a non-clinical sample only when at least one copy of the D4 dopamine receptor gene (DRD4) 7R allele was present (84). Another study found that BDNF Val66Met polymorphisms moderate the interactive effects between 5-HTTLPR and stress on HPA axis reactivity in a sample of preschool children (85). Therefore, it seems necessary to include several genetic polymorphisms within crucial pathways of stress regulation and investigate their interactive effects in more detail.

In a seminal study, Tyrka et al. (86) evaluated whether the functional polymorphisms rs110402 and rs242924 in the CRHR1 gene, which previously had been identified as an important moderator of depression risk after ELS [see above; (25)], interacts with child maltreatment to predict HPA axis reactivity. For each polymorphism, subjects who carried the GG genotype and who reported a history of child maltreatment exhibited elevated cortisol responses in the dexamethasone/CRH test. In accordance with the clinical outcome study (25), A allele carriers (AA, AG) with child maltreatment experience were protected and showed responses similar to persons with no child maltreatment experience. In persons with no child maltreatment experience, CRHR1 polymorphisms did not modify HPA axis reactivity. These results suggest an interaction effect, in which the CRHR1 gene and childhood maltreatment impact on glucocorticoid-mediated inhibition of the HPA axis under challenge conditions, as tested by the specific test. Increased responsiveness in the dexamethasone/CRH test has long been known to be a potent risk marker for depression (87). Hence, this study provides important insight into the potential mechanism for a Gene × ELS interaction in depression.

A study published in the same year by Heim et al. (22) replicated and extended this finding. In this study, it was shown that the effect of CRHR1 rs110402 in moderating depression risk in relation to ELS exposure was limited to men, but could not be observed in women; this apparent sex difference was attributable to differential exposure types between the sexes, with men experiencing more physical abuse than women (see above). Of note, in response to the same challenge test used in the study reported in Ref. (86), there was a graded effect of the protective A allele on HPA axis reactivity (GG > AG > AA) that was observed only in male participants, whereas the polymorphism had no effect on cortisol response in women. Accordingly, women with maltreatment showed enhanced cortisol responses to the dexamethasone/CRH test, whereas maltreated men did not. These results demonstrate that the complex interplay of abuse type, sex, and genetic variation is reflected at the level of an intermediate phenotype that is a relevant risk marker for depression. Cicchetti et al. (30) recently extended to this line of evidence showing that the CRHR1 TAT haplotype interacts with 5-HTTLPR to predict diurnal cortisol levels in maltreated children.

The GR is critically implicated in regulating HPA axis reactivity and, as noted above, a polymorphism in the GR gene moderates the link between ELS and depression (33). In the same study, it was shown that the 22/23EK polymorphism in the GR gene interacts with ELS in predicting a free cortisol index, calculated as ratio between total cortisol and cortisol binding globulin levels. In the light of evidence that the FKBP5 gene moderates risk for affective disorders in relation to ELS [see above; (35–37)], several studies investigated interactions of FKBP5 gene variants and stress in determining indices of HPA axis function. Ising et al. (88) report that three FKBP5 polymorphisms are associated with prolonged cortisol elevations after standardized psychosocial stress (rs4713916: GG > GT,TT; rs1360780: TT > CT,CC; rs3800737: AA > AG,GG), although ELS was not assessed in this study. Effects of FKBP5 polymorphisms on dexamethasone-induced suppression of cortisol have been reported in interaction with child abuse-related PTSD, inasmuch as risk variants in various FKBP5 polymorphisms interacted with PTSD status in predicting hyper-suppression of cortisol (75, 89). These findings show that risk genes may interact with a clinical diagnosis that is in turn related to ELS; however, similar results for depression are lacking. In a recent seminal study (90), the same group provides strong evidence for an FKBP5 × ELS interaction in determining de-methylation of functional glucocorticoid response elements (GREs) in the FKBP5 gene. This de-methylation was associated with an increased transcription of the gene, leading to enhanced feedback between FKBP5 and GR and, consequently to GR resistance. Effects of this risk allele-specific and ELS-dependent de-methylation were shown at the level of *in vitro* glucocorticoid sensitivity, glucocorticoid-regulated metabolic and immune gene expression, as well as brain structure (90). This remarkable study is the first to map in detail a molecular to neural pathway that underlies a Gene × ELS interaction effect in the prediction of depression risk.

Other studies have investigated interactions of ELS and candidate genes implicated in stress or regulation, although these candidate genes have not been considered in Gene × ELS studies predicting clinical depression outcomes. Neuropeptide Y (NPY) is a peptide that is known to exert anxiolytic and stress-buffering effects in the central nervous system. A polymorphism in the NPY gene promoter, rs16147, was found to interact with ELS in predicting neuroendocrine response to psychosocial laboratory stress in a prospective cohort study of children at risk conducted in Germany (91). A study by Armbruster et al. (92) focused on the enzyme, catechol-*O*-methyltransferase (COMT), which catabolizes catecholamines. Results revealed main effects for COMT and stressful life events, but no interaction effect between the two factors, suggesting that the COMT gene operates independently from stress exposure and is likely not involved in mediating the link between ELS and depression.

### Gene × ELS interaction effects on regional brain structure and function

The study of the impact of specific serotonin genotypes on structural and functional characteristics of the brain related to depression has become of great interest in recent years. In this context, the prefrontal–amygdala interaction seems to be of particular importance in affective processing and was found to be differentially affected by 5-HTTLPR genotype in depressive patients and healthy controls (93). These findings were further specified in a study on healthy adults (94). In this study, carriers of the risk s-allele displayed increased amygdala activation. Further statistical analysis revealed that this genotype effect was based on the amygdala structure, which proved to be smaller in the s-allele carriers compared to the LL genotype. These findings indicate that genetic effects during neurodevelopment play a role in this context.

At a structural level, serotonin 3A receptor genotype and its interaction with ELS were investigated with regard to fronto-limbic gray matter in a non-clinical sample (95). Interestingly, the HTR3A CC genotype group displayed gray matter loss in hippocampal structures compared to the TT genotype group, with CC carriers plus ELS showing additional gray matter loss in frontal cortices. In line with this, another study found that carriers of 5-HTTLPR risk s-allele displayed smaller hippocampal volumes following a history of emotional neglect in depressive patients compared to patients without ELS or genetic risk genotype (62). Independent of genotype, hippocampal white matter alterations were predicted by childhood stress in patients. Compared to healthy controls, the PFC was smaller in patients. Of note, patients with ELS and non-risk l-allele genotype displayed a larger PFC, indicating compensatory or protective characteristics. A recent study in children further highlights the potential mechanisms of Gene × ELS interactions (96). Study participants who were carriers of the 5-HTTLPR s-allele showed greater attentional bias to subliminally presented fearful faces than carriers of the non-risk l-allele. The former also showed increased neural activations to fearful and angry faces in regions of the association cortex linked to attention-control in adults. The s-allele genotype, therefore, seems to be related to hypervigilant behavioral and neural activation in face of negative stimuli. In the same line, the effects of acute stress were investigated in a group of healthy women (97). Women with the SS genotype displayed increased neural activation during threat anticipation in a large corticolimbic network compared with l-allele carriers, potentially reflecting increased sensitivity to develop psychopathological symptoms when faced with stressful events. In healthy men, similar Gene × ELS interactions were found regarding neural response patterns and functional amygdala–hypothalamus connectivity (98). S-allele carriers with a history of ELS showed heightened sensitivity toward negative, fearful stimuli in this context. These findings were further supported by a study, which focused on fear conditioning in a similar experimental setting (99). Another recent study found an interaction between recent stressful life events and SNPs within the HTR1A and HTR1B genes regarding symptom scores of depression (100). More specifically, the interactive effect was observed with regard to HTR1A rs878567 T alleles and rs6295 G alleles as well as HTR1B rs6296 C and recent stressful life events. An experimental face emotion processing task was presented to a subgroup of the population sample to further investigate threat-related information processing. Notably, healthy HTR1A rs6295 GG carriers did not display an affective bias to perceive more negative emotions but showed reduced reaction times when presented with fearful faces. This modulation of threat-related information processing by genotype may predispose certain individuals to develop depression throughout the life course.

As mentioned earlier, the CRH system plays an important role in neuroendocrine and behavioral responses to stress and is regarded as a central component within the pathogenesis of depression. Especially polymorphisms of the CRHR1 gene seem to play an important role in moderating the effects of stress. A recent study found that variations in the CRHR1 gene, more specifically SNP rs110402, moderate neural responses to emotional stimuli (101). A differential pattern of results emerged between A allele carriers and GG homozygotes suggesting that vulnerability to depression is associated with a specific CRHR1 genotype.

Reward learning was in the focus of a study regarding the effects of the CRHR1 SNP rs12938031 and acute stress (102). Reward learning is regarded as a crucial component of anhedonia, which is closely related to stress-related psychopathology. The authors investigated response bias as a function of reward in healthy female participants under no-stress and acute stress conditions. The authors found that acute stress led to reduced response bias, which indicates impaired reward learning under stressful conditions. Notably, an interaction between rs12938031 and acute stress was also observed, with A homozygotes displaying stress-related impairment in reward learning. Behavioral findings were also supported by neural activation patterns. The modulation of reward learning by genotype may lead to increased vulnerability to depression in some individuals.

Another recent study investigating this issue used the personality trait neuroticism as proxy for depression given that it reflects a tendency to experience negative affect and that it is a major risk factor for developing depression (103). As childhood maltreatment is also regarded as major risk factor for depression, the authors were interested in investigating the interaction of childhood maltreatment with a specific genotype regarding the personality trait of neuroticism. Based on previous research findings, variation in the three CRHR1 SNPs rs110402, rs2452924, and rs7209436 was investigated in maltreated as well as in non-maltreated children who participated in a day camp research program. Number and types of stressors were also taken into account in the analysis. As expected by the authors, a significant interactive effect between childhood maltreatment and CRHR1 genotype was found in the prediction of neuroticism. Maltreated children with two copies of the TAT haplotype of CRHR1 showed higher levels of neuroticism than non-maltreated children with the same genotype. Notably, this effect was not displayed in children with multiple types of maltreatment or sexual abuse, who seemed to be protected from increased neuroticism by the same genotype. This remarkable finding needs to be explored in further detail and highlights the importance of distinguishing between different types and numbers of stressors.

The BDNF gene polymorphism Val66Met has been found to be related to anxiety and mood disorders (104). More specifically, the interaction between BDNF Val66Met polymorphism and ELS was investigated with regard to brain and arousal pathways to syndromal depression and anxiety in a non-clinical sample. Significant interactions between BDNF genotype and ELS emerged with BDNF Met-carriers exposed to greater ELS showing smaller hippocampal and amygdala volumes, heart rate elevations, and a decline in working memory. The relationship between this polymorphism and specific, emotion-related brain circuitries has also been studied in anxious and depressed adolescents compared to healthy controls (105). In the patient group, a significant interaction between genotype and emotional stimulus presentation was found. Met-carriers showed greater neural responses than Val/Val homozygotes when presented with emotional faces.

The MR iso/val polymorphism (rs5522) has recently been found to moderate the association between childhood emotional neglect and amygdala reactivity in children and adolescents (106). The interactive effects of childhood maltreatment and the OXTR SNP rs53576 on adult emotional dysregulation and attachment style were investigated in a sample of low-income, African American men and women (107). Significant interactive effects were found between childhood maltreatment and OXTR rs53576 in predicting levels of adult emotional dysregulation and attachment style. While G/G allele carriers appeared to be particularly vulnerable to severe childhood maltreatment, A/A and A/G allele carriers displayed resilience. Distinguishing between numbers and types of stressors was crucial in this study. Other findings indicate that NPY genetic variation may predispose to low NPY expression, thereby increasing neural responsivity to negative stimuli in emotion-related brain circuitries (108).

Gene × Gene × ELS effects also need to be considered in this context as recent studies have found interactive effects for 5-HTTLPR and BDNF, CREB1, BDNF and NTRK2, as well as BDNF and HTR3A genotypes (109–111). CREB1 (cyclic adenosine monophosphate response element-binding protein 1), BDNF and NTRK2 (neurotrophic tyrosine kinase receptor, type 2) form a neuroplastic pathway, which plays a crucial role in brain adaptation to stress (110).

## Conclusion

One of the most promising pathways of future research is the investigation of epigenetic processes modulating the relationship between Gene × ELS interactions and depression. In this context, quality of maternal care was found to influence hypothalamic–pituitary–adrenal function in rats through epigenetic programing of GR expression (112). Low levels of maternal care were associated with increased methylation of the promoter region of the GR gene as well as enhanced hormonal and behavioral responses to stress. These findings could be translated to the brains of human suicide victims who had experienced childhood abuse compared to those victims who had not (74) and were further reflected in a study on childhood adversity and epigenetic modulation of the leukocyte GR (75). Adding to these findings, differential maternal rearing conditions led to differential DNA methylation in PFC and T cells of rhesus macaques (113). These findings could be further translated and expanded by evidence suggesting that the trajectory of male physical aggression is associated with differential DNA methylation in cytokines and their regulators in T cells and monocytes (114). The seminal findings with regard to epigenetic processes and FKBP5 following childhood trauma have substantially added to this knowledge and will encourage future research endeavors (90).

It is important to note that past studies on Gene × ELS interactions and depression have been subject to a large amount of heterogeneity due to differences in experimental procedures. An important difference concerns the measurement of ELS, with critical issues concerning timing of measurement, type, and context of ELS studied as well as gender-specific considerations (115). In addition, timing of onset of depression and chronicity of the disorder may be an important factor in this context and should be considered in future research efforts.

Previous findings with regard to Gene × ELS interactions and depression suggest that genes and environment have significant interactive effects in predicting depression, in the absence of strong main genetic effects (3). While previous research has been hypotheses-driven focusing on candidate gene approaches, future studies will move beyond this confinement to genome-wide hypotheses-free studies, which require much larger study samples. By this means, new patterns of gene classes or transcription factor binding sites, which are altered by ELS, may be detected, thereby supporting the current strive for adequate prevention and treatment schemes in sufferers from childhood trauma.

Future research will further investigate the differential role of certain genotypes as a function of adverse and beneficial environments. Current models on childhood development outline the concept of a differential susceptibility to environment, i.e., some individuals are more susceptible than others to both negative (risk-promoting) and positive (development-enhancing) environmental conditions (116). Recent findings on Gene × ELS interactions seem to confirm the concept of differential susceptibility (107, 117), which questions the classic diathesis–stress framework as conceptual basis of past research. In this context, the identification of developmentally sensitive periods of brain maturation will be of particular importance when it comes to prevention and early intervention (118).

The existence of biologically distinguishable subtypes of depression as a function of ELS is of importance in the response to differential treatment (59). When comparing the effectiveness of pharmacotherapy and psychotherapy in the treatment of depressed patients with and without childhood trauma distinct patterns of treatment success were observed (119). In absence of early trauma, combination treatment proved to be the most effective treatment option, whereas in case of early trauma, remission rates were higher for psychotherapy than for pharmacological treatment and combination therapy did not lead to any advantage over the effects of psychotherapy alone. These differential effects may be based on differences in neurobiological pathways to depression, which need to be further elucidated (120).

Taken together, the latest research findings have underscored the essential role of genotypes and epigenetic processes within the development of depression after childhood trauma, thereby building the basis for future research and clinical interventions.

## Conflict of Interest Statement

The authors declare that the research was conducted in the absence of any commercial or financial relationships that could be construed as a potential conflict of interest.
